# Genipin-activating PPARγ impedes CCR2-mediated macrophage infiltration into postoperative liver to suppress recurrence of hepatocellular carcinoma

**DOI:** 10.7150/ijbs.87327

**Published:** 2023-10-16

**Authors:** Junyu Wu, Yau-Tuen Chan, Yuanjun Lu, Zixin Feng, Hongchao Yuan, Xiaoyu Xu, Lin Xu, Cheng Zhang, Yibin Feng, Hor-Yue Tan, Ning Wang

**Affiliations:** 1 School of Chinese Medicine, The University of Hong Kong, Hong Kong S.A.R., China.; 2 Centre for Chinese Herbal Medicine Drug Development, School of Chinese Medicine, Hong Kong Baptist University, Hong Kong S.A.R., China.; 3 Department of Chinese Medicine, the University of Hong Kong-Shenzhen Hospital (HKU-SZH), Shenzhen, China.

**Keywords:** HCC, postoperative recurrence, macrophage, chemotaxis, PPARγ, genipin

## Abstract

A high postoperative tumour recurrence rate has significantly rendered a poorer prognosis in hepatocellular carcinoma (HCC) patients. The aim of this study is to identify a natural compound genipin as a potential and effective candidate to suppress the postoperative recurrence of HCC. Clinical analysis revealed that infiltration of macrophage into the adjacent tissue but not HCC predicted patients' poor prognosis on recurrence-free survival. Genipin intervention suppressed the Ly6C+CD11b+F4/80+ pro-inflammatory macrophage infiltration in the postoperative liver of mice. Adoptive transfer of pro-inflammatory monocytic cells completely abolished the inhibitory effect of genipin on HCC recurrence. Transcriptomic analysis on FACs-sorted macrophages from the postoperative livers of mice revealed that PPARγ signalling was involved in the regulatory effect of genipin. Genipin is directly bound to PPARγ, causing PPARγ-induced p65 degradation, which in turn suppressed the transcriptional activation of CCR2 signalling. PPARγ antagonist GW9662 abrogated the effects of genipin on CCR2-medaited macrophage infiltration as well as HCC recurrence. Cytokine array analysis identified that genipin intervention potently suppressed the secretion of CCL2 further partially contributed to the pro-inflammatory macrophage infiltration into the postoperative liver. Multiplex immunostaining on tissue array of human HCC revealed that PPARγ expression was inversely associated with CCL2 and the macrophage infiltration in the adjacent liver of HCC patients. Our works provide scientific evidence for the therapeutic potential of genipin as a PPARγ agonist in preventing postoperative recurrence of HCC.

## Introduction

Hepatocellular carcinoma (HCC) is one of the deadliest cancers and has caused a great threat to the global healthcare systems 1, 2. For most patients with the early or intermediate stage of HCC, surgical resection remains the mainstay treatment. However, the prognosis of patients receiving hepatectomy is significantly compromised by the presence of postoperative recurrence, the 5-year incidence of which has reached as high as 70% 3, 4. Patients who developed intra-hepatic tumour relapse were associated with a significantly lower 3- and 5-year overall survival rate (36.6% and 6.1% vs. 62.9% and 13.8; p<0.001) as compared with non-recurring counterparts 5. Generally, HCC recurrence is further divided into early and late recurrence based on the cut-off of two years after surgery 6 . While early recurrence is believed mainly derived from refractory intrahepatic metastasis, late recurrence is mainly attributed to *de novo* carcinogenesis 7. Patients with early recurrence have a significantly worse prognosis in comparison with the late counterpart (27.1%, 61.9% vs. 4.5%, 25.7% at 3- and 5-year survival rates) 8. To date, repeated hepatectomy and salvage liver transplantation are the clinically approved treatment options for recurrent HCC. However, repeated hepatectomy is less effective and liver transplantation is subject to the donor source 9. There is no other approved systemic treatment for preventing HCC recurrence after the failure of sorafenib in a phase III trial, underlining the significance of developing neoadjuvant postoperative therapeutics 10.

The tumour progression and recurrence in HCC requires a highly orchestrated network compromised by immune and stromal components to support the nutritional demands and proliferation of malignant cells. After partial hepatectomy, the intrahepatic metastasis that is left deep in the remnant liver takes advantage of abundant growth factors and cytokines brought by liver regeneration and postoperative inflammation to rapidly expand, which to some extent explains why the tumour progresses at a faster pace after surgery 11, 12. Hence, the postoperative tumour microenvironment (TME) should be considered as a target in treating HCC early recurrence 13, 14. Among the immune TME, the macrophage is one of the major immune populations that exert multiple pro-tumoral functions during primary HCC progressions such as promoting angiogenesis and immunosuppression 15-17. High infiltration of macrophages in primary HCC and peritumoral tissues were associated with a worse prognosis after tumour resection 18-20. Inhibiting macrophage influx by CCR2 antagonist efficiently suppressed HCC recurrence in a mouse model 21. All these collectively suggest postoperative macrophage infiltration predisposes to HCC recurrence.

Genipin is a natural iridoid aglycone derived from the Chinese herbal Medicine *Gardenia jasminoides*, which has been commonly used in the clinical treatment of inflammation-associated diseases and heat-related liver cancer 22-25. Our previous studies previously observed that oral administration of genipin reduced the risk of lung metastasis of HCC cells in the experimental mouse model of HCC 26, 27. However, whether genipin decelerates postoperative HCC recurrence remains to be defined. In this study, we utilized the orthotopic mice model with partial hepatectomy to evaluate the effect of genipin on HCC recurrence, and identified genipin as a promising therapeutic agent for the treatment of HCC recurrence through targeting postoperative macrophage influx.

## Materials and methods

### Ethics statement

All animal experiments were conducted according to the protocols approved by the Committee on the Use of Live Animals in the Teaching and Research of the University of Hong Kong (CULATR 5242-19).

### Animals and orthotopic HCC tumour implantation model

The C57BL/6 mice, BALB/c nude mice and NOD-SCID mice (male, 4-6-week-old) were provided by the Centre for Comparative Medicine Research of the University of Hong Kong. Two types of orthotopic HCC mouse models were established. The first type of orthotopic HCC mouse model was established as described in our previous study.^28^ Briefly, luciferase-expressed MHCC-97L cells (1 x 10^6^) were subcutaneously injected into the right flank of a NOD-SCID mouse. The tumour was isolated when the diameter reached 10 mm and then cut into small cubes (around 1 mm^3^). Subsequently, the tumour cube was implanted in the left lobe of the liver of the BALB/c nude mouse. The growth of the orthotopic tumour was monitored by capturing the bioluminescence signal by a Xenogen IVIS imaging system (PerkinElmer) once a week. Another immunocompetent orthotopic HCC tumour model was constructed by injection of 100 μL 1 x 10^6^ Hepa 1-6 cell suspensions into the left lobe of the liver of C57BL/6 mice.

### Surgical resection of orthotopic tumours (partial hepatectomy)

Fourteen days after orthotopic tumour implantation, mice bearing orthotopic liver tumours were anaesthetized and subjected to tumour resection. An incision was made in the middle upper abdomen, and the liver lobe implanted with the tumour was visualized and excised. The distance between the excisional margin and the tumour edge was set to be greater than 2 mm. Mice with peritoneal tumour adhesion were excluded from partial hepatectomy and subsequent experiments. The successful resection of primary tumours was confirmed by bioluminescence signal analysis with the Xenogen IVIS imaging system (Perkin Elmer). The onset and progression of postoperative recurrence were monitored and quantified by the bioluminescence signal detected by the Xenogen IVIS imaging system.

### Histology and immunofluorescence

The collected tissues were fixed with 4% formalin buffer (PFA) and then subjected to paraffin embedding. The samples on the slide (5 µm) were dewaxed by xylene and rehydrated by gradient ethanol (100% ~ 50%). For H&E staining, slides were incubated with haematoxylin for 5 min and then with 0.25% eosin for another 5 min. The slides were subsequently soaked in Canada balsam (Sigma-Aldrich, USA) and imaged using a BX43 optical microscope (Olympus, Japan). For immunofluorescence staining, after rehydration, antigen retrieval was performed with a 10 mM citric acid buffer and then blocked with 10% goat serum at room temperature for 1 h. The slides were incubated overnight with the primary antibodies Ki67 (ab15580, Abcam) and F4/80 (ab6640, Abcam) at 4°C, followed by secondary antibody Alexa Fluor 568 (A-11031, Invitrogen) for 1 h. Nuclear staining was performed by DAPI (D1306, Invitrogen). Finally, the slides were mounted with a fluorescent mounting medium (Dako, Denmark). Images were captured by confocal microscope (Carl Zeiss LSM 980/900, Germany).

### Cell culture

Luciferase-tagged MHCC-97L cells were obtained from Prof. Man Kwan from the Department of Surgery, at the University of Hong Kong. AML-12 cells were purchased from ATCC and cultured in complete DMEM/F12 medium supplemented with 10% fetal bovine serum and 1% penicillin/streptomycin.

### Fluorescence-activated cell sorting and Flow cytometry

For *in vivo* immune profile analysis, single-cell suspensions were obtained by digesting an equal amount of postsurgical liver tissues using Collagenase, Type IV (Thermo Fisher). Peripheral blood mononuclear cells (PBMC) were isolated from mouse peripheral blood by the Lymphoprep (STEMCELL Technology, Canada). Cell cultures were collected by trypsinization. The suspended cells were passed through a 70 µm strainer and resuspended in 0.1% BSA PBS. The cells were blocked by CD16/32 antibody (BioLegend) for 5 min before antibody staining at room temperature for 15 minutes. The flow cytometry analysis was performed on the NovoCyte Quanteon analyzer (Agilent Technologies), and fluorescence-activated cell sorting was performed on BD FACSAria^TM^ Fusion Cell Sorter (BD Sciences). All antibodies used for flow cytometry are listed in Table S1.

### Isolation of bone marrow-derived mononuclear cells

Bone marrow-derived mononuclear cells (BMDMs) was harvested from femurs and tibias of C57BL/6 mice (6-8-week-old) and separated by the Lymphoprep (STEMCELL Technology, Canada). BMDMs were subsequently cultured in RPMI-1640 (supplemented with 10% fetal bovine serum and 1% penicillin/streptomycin) and stimulated with 10ng/ml mCSF (576404, Biolegend).

### Macrophage adoptive transfer

BMDMs were isolated and stimulated as described above. The macrophage adoptive transfer was conducted by intraperitoneal injection of 5× 10^6^ BMDMs to the postsurgical mice every 3 days starting from day 3 after partial hepatectomy.

### PKH26 labelling

BMDMs were isolated from tumour-bearing mice as previously described. The neutrophils were resuspended at a concentration of 1 × 10^7^ cells/mL in serum-free RPMI-1640 medium and stained with 2.5 µmol/L PKH26 dye (Sigma-Aldrich) for 5 min. These PKH26 labelled cells were intraperitoneally re-supplemented to the postsurgical mice with or without genipin treatment. After 24h of injection, the postoperative liver tissues were collected, and the proportions of migratory macrophages were analyzed using flow cytometry.

### Cytokine array

Mouse cytokine array was performed using C-Series Mouse Cytokine Antibody Array C2000 (RayBiotech, USA) according to the manufacturer's manual. Membrane exposure was performed using a BioRad ChemiDoc XRS+ imaging system (BioRad Laboratories) and analyzed by ImageJ (NIH, USA).

### Macrophage chemotaxis assay

The *in vitro* macrophage chemotaxis assay was performed by transwell migration assay. BMDMs were pre-treated with a series concentration of genipin for 48 h and resuspended in a serum-free DMEM medium. 200 µl cell suspension containing 5 x 10^4^ cells were seeded into the upper chamber of a 24-well plate. The lower chamber was filled with 800 µL DMEM supplemented with 40ng/ml mouse recombinant CCL2 (578404, Biolegend) or AML-12 conditioned medium. After 48 h incubation, the cell remaining in the upper chamber was removed with a cotton swab. The cells at the bottom surface of the chamber were fixed with 4 % PFA for 20 min at room temperature. Subsequently, cells were stained with 0.1 % crystal violet for 30 min and washed with tap water. The chamber was dried at 37°C and the cells were imaged by a microscope (EVOS, USA). The number of migrated cells was quantified by ImageJ.

### Cell apoptosis detection

Pharmingen annexin V-FITC apoptosis detection kit (BD) was utilized to detect cell apoptosis. Briefly, BMDMs were treated with different concentrations of genipin for 24 h. Subsequently, cells were harvested and stained with PE-annexin V and 7-AAD for 15 min at room temperature. Flow cytometry (Agilent NovoCyte Advanteon BVYG) analysis was employed to measure cell apoptosis.

### Phagocytosis analysis

pHrodo™ Green E. coli BioParticles™ Conjugate (Thermo, USA) was utilized to detect the phagocytosis of BMDMs. Briefly, BMDMs were treated with different concentrations of genipin for 24 h before subject to 50 ug/ml pHrodo™ Green E. coli BioParticles™ Conjugate incubation at 37°C for 2 hours. Finally, cells were harvested and subject to flow cytometry analysis.

### High throughput RNA sequencing and real-time quantitative PCR

The total RNA of macrophages sorted from the tumour microenvironment of HCC in mice with or without genipin treatment was extracted using Trizol (Takara, Japan) according to the manufacturer's instructions. For RNA sequencing, the total RNA of Ly6C^+^CD11b^+^F4/80^+^ cells (n = 3) were submitted to Novogene (Beijing, China) for analysis. For reverse transcription-PCR (RT-PCR), PrimeScript RT master mix (Takara, Japan) was utilized for cDNA first-strand synthesis. SYBR green Probe (Takara, Japan) and specific primers were used for RT-PCR on a Light Cycler 480 PCR system (Roche, Switzerland). The sequences of primer sets are listed in Table S2.

### Western blot

Cells and tissues were lysed on ice for 30 min using RIPA buffer (Thermo Fisher Scientific, USA) supplemented with proteinase/phosphatase inhibitor (#5872, Cell Signaling Technology). The protein lysates were collected after 12,000 rpm centrifugation for 10 min at 4°C. Bradford assay was used to quantify the concentration of protein lysates. The same amount of protein was separated by SDS- PAGE and transferred to the PVDF membrane. Subsequently, the membranes were incubated with 5 % BSA for blocking for 1 h at room temperature and then incubated with primary antibodies at 4°C overnight. Secondary antibodies were incubated at room temperature for 1 h. Signals were detected with ECL advanced kit (GE Healthcare) using a chemiluminescence imaging system (Bio-Rad). The primary antibodies: β -ACTIN (AC026), PPARγ (A0270) and NF-kB p65/RelA (A19653) were purchased from ABclonal (USA).

### Chromatin immunoprecipitation (ChIP)-qPCR

ChIP was performed by following the protocol of the EZ-Magna ChIP A/G Chromatin Immunoprecipitation kit (Sigma-Aldrich). AML-12 cells and BMDMs (1 x 10^7^) treated with 40 μM genipin or vehicle for 48 h were fixed with 1 % PFA and then washed by PBS. The cells were collected and subjected to cellular and nuclear lysis. The whole nuclear lysate was sheared by a sonicator with optimal conditions (7 s pulse on, 10 s pulse off, 15 cycles, 40 % amplitude) to yield 200-700 pb DNA. 10 % of sheared lysate was used for the Input. 50 μL of the sheared lysate (the equivalent of 1 x 10^6^ cells) was subject to immunoprecipitation by overnight incubation of either anti-NF-kB p65/RelA antibody (A19653, Abclonal), anti- PPARγ (C26H12, Cell Signaling) or IgG control. The immunoprecipitated DNA and Input DNA were purified and amplified by RT-PCR with primers sets listed above.

### Coimmunoprecipitation

Aml-12 and BMDMs treated with genipin or vehicle were lysed by NP‐40 cell lysis buffer (Life Technologies, USA). The target protein was precipitated with NF-kB p65/RelA Rabbit mAb (A22331, Abclonal) coupling to Protein A/G magnetic beads (HY-0202. MCE), which was followed with the elution (TBS in 0.1% Tween 20). The specific protein-protein complex was pulled down and separated by SDS‐PAGE. The chemiluminescence system (Bio‐Rad, USA) was applied for the visualization and quantification of the target proteins.

### Multiplex immunohistochemistry

Multiplex IHC was performed as described previously 29. The antibody panel was listed in Supporting Table S3. The multiplex IHC was conducted on the tissue microarray, which incorporates 90 HCC samples with their paired adjacent liver tissues. The patient's clinical information was listed in Supporting Table S4. The IHC staining reagents were bought from AKOYA BIOSECIENCES (NEL797B001KT, PerkinElmer, Massachusetts, USA). Briefly, the tissue slide was dewaxed, rehydrated, fixed in 10% neutral buffered formalin and subjected to epitope retrieval by boiling in AR6 buffer for 15 min. Antibody Diluent/Block reagent was utilized to block the endogenous peroxidase. In each round of IHC staining, one antigen was labelled with one corresponding fluorophore and the primary-secondary-HRP complex was eluted by boiling in AR6 buffer. PPARγ, CCL2, TIM-4, ZO-1, CD31 and CD68 were stained with fluorophores Opal 480, Opal 520, Opal 570, Opal 620, Opal 650 and Opal 780 respectively. After the staining of 6 antibodies, the slide was counterstained with DAPI and enclosed in the mounting medium for subsequent imaging. The slide without primary antibody incubation served as negative control and was also subjected to the detection of autofluorescence. The imaging was performed on Vectra Polaris (PerkinElmer, Massachusetts, USA). The 7 multispectral dyes were unmixed in the Inform Advanced Image Analysis software (inForm 2.4.0; PerkinElmer, Massachusetts, USA) using library slides.

The quantification of multiplex IHC was evaluated using inForm software. We first performed cell segmentation to segment major cell components including the nuclei, cytoplasm and membrane. Then a learning algorithm was adopted to recognize and quantify different types of cells. For each type of cell, we manually identified 20-30 typical cellular phenotypes as examples for the subsequent classifier training, which will automatically generate a cell phenotype map on the whole tissue section.

### Statistical analysis

All statistical analysis was performed by GraphPad Prism 9 software (CA, USA). Two-tailed Student's unpaired t-test was used for two-group comparison and One-way ANOVA was utilized for multi-group comparison, respectively. All experiments are conducted independently three times or above. All data are presented as mean ± SD. *P* value < 0.05 was considered to be statistically significant.

## Results

### Macrophage infiltration in the adjacent liver but not tumour tissues predicted poor prognosis of HCC after surgical resection

**Figure 1:**
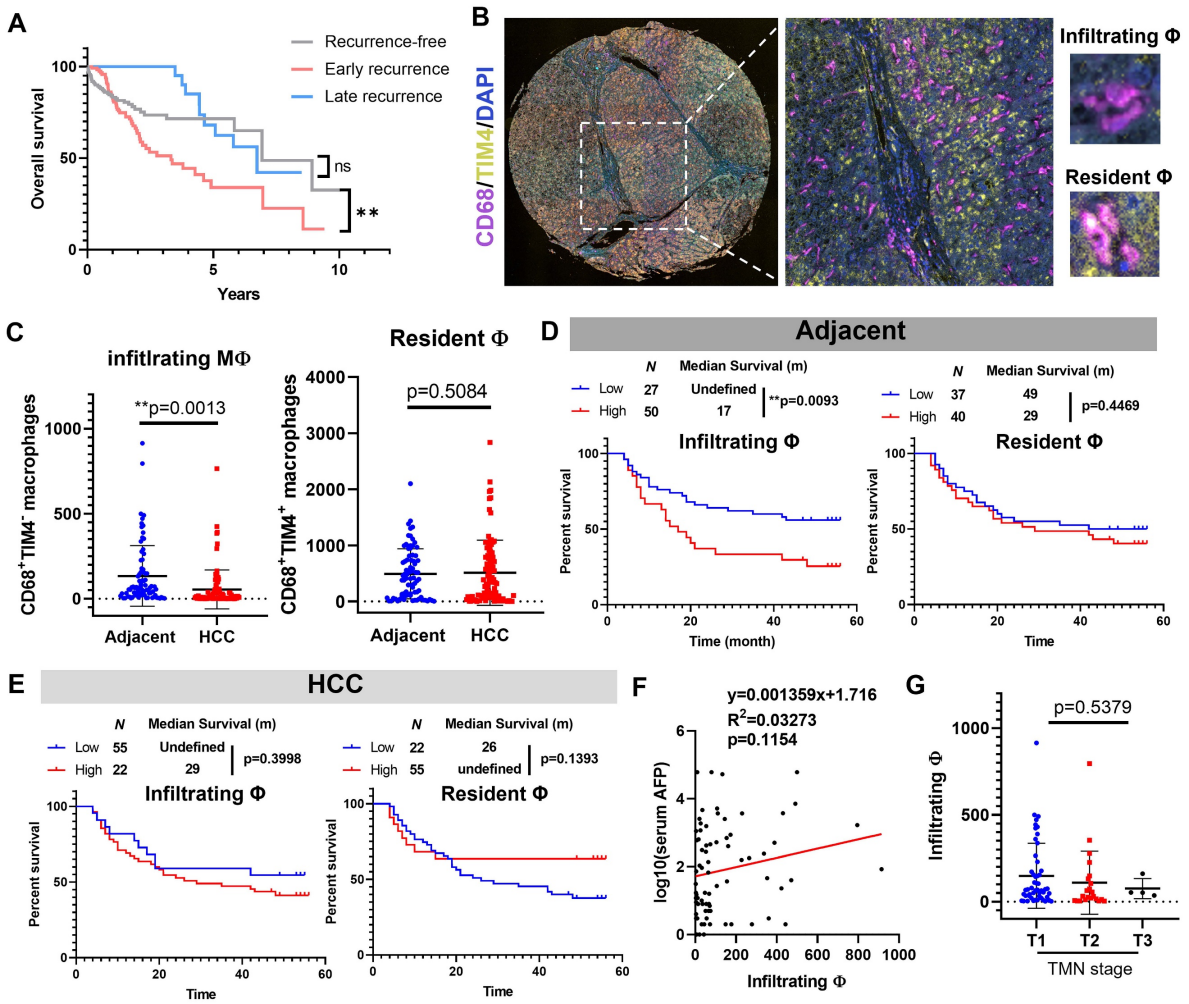
** Infiltrating macrophages in adjacent tissue but not HCC predicted poor prognosis of patients. A.** Kaplan-Meier survival plots showing the overall survival results of recurrence-free HCC patients, early recurrent HCC patients, late recurrent HCC patients. **B.** Representative image of TIM-4^-^CD68^+^ monocyte derived macrophages (MDM) and TIM-4^+^CD68^+^ tissue resident macrophages in adjacent liver tissues. **C.** Adjacent tissues showed high infiltrating macrophages than HCC tissues, while resident macrophages remained similar. **D.** Infiltrating but not resident macrophages in adjacent tissue predicted poor prognosis of the recurrence-free survival of HCC patients. **E.** Neither infiltrating nor resident macrophages in HCC was associated with the recurrence-free survival of HCC patients. **F&G.** infiltrating macrophages in the adjacent tissues was not related to the serum AFP level or TMN stage of HCC patients. *p < 0.05; **p < 0.01; ***p < 0.001.

We first retrieved data from TCGA-LIHC cohort and plotted their Kaplan-Meier curves based on their recurrence status. Patients with late recurrence have a highly similar prognostic performance with recurrence-free patients, while early recurrence significantly undermines the survival of HCC patients (Fig. 1A), which suggested inhibiting or decelerating-postoperative-early-recurrence were crucial for expanding HCC patient's survival after surgical resection. To explore the clinical significance of hepatic macrophages in the HCC recurrence of patients, we performed multiplex immunohistochemical staining on a tissue array from 78 patients containing HCC sections and their paired adjacent tissue sections. CD68 was used to recognise hepatic macrophages, and we assigned TIM4 as the cellular marker to differentiate infiltrating macrophages from their resident counterparts (Fig. 1B). By virtue of a training-based algorithm, we acquired the phenotype of all cells appearing in each tissue section, which were further quantified and integrated for clinical significance analysis. Surprisingly, we observed a higher level of infiltrating macrophages in the adjacent liver tissue compared to its paired HCC tissues, while the resident macrophages remained no differences (Fig. 1C). Further analysis plotting the recurrence-free survival of HCC patients showed that high level of infiltrating macrophages in the adjacent tissues predicted poor prognosis of HCC patients after surgical resection, while the resident macrophages in adjacent tissues showed minimal impact on patients' recurrence-free survival (Fig. 1D). Neither infiltrating nor resident macrophages in the HCC tissue could effectively predict the recurrence-free survival of the patients (Fig. 1E). The infiltrating macrophages in the adjacent tissue of HCC patients was not associated with serum AFP level or TMN staging (Fig. 1F&G). These observations suggested that Macrophage infiltration in the adjacent liver but not tumour tissues predicted poor prognosis of HCC after surgical operation.

### Genipin suppressed HCC recurrence and macrophage infiltration into the liver after partial hepatectomy

**Figure 2:**
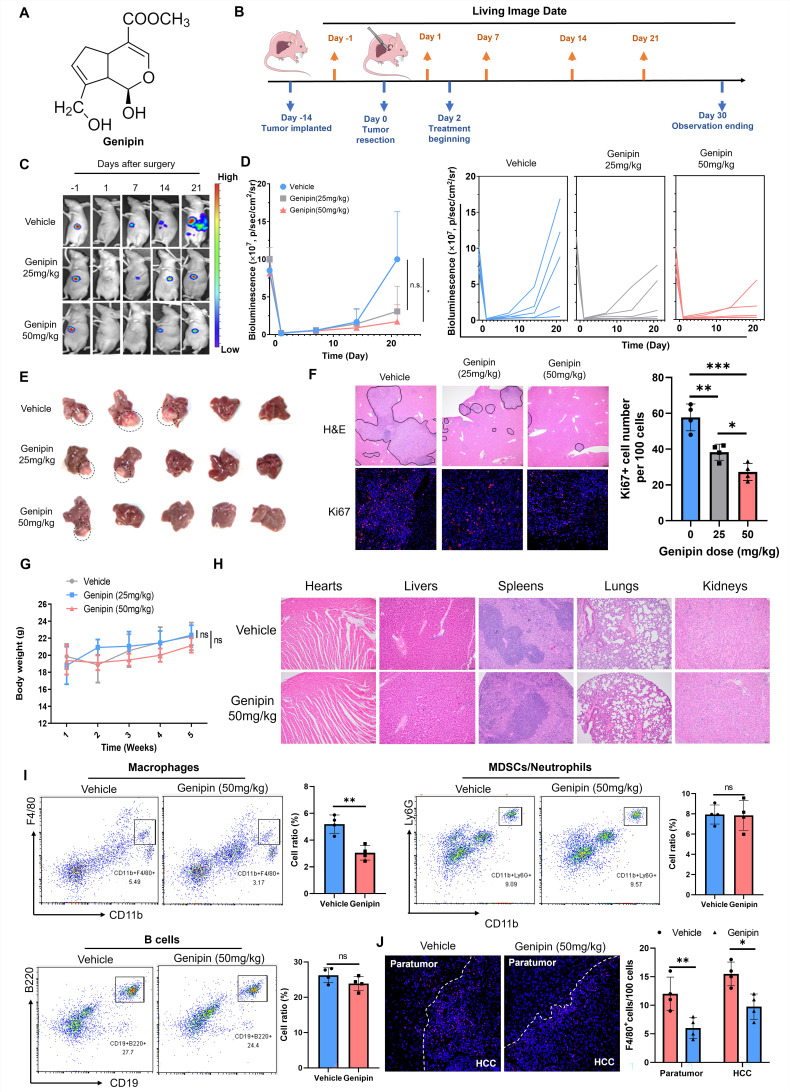
** Genipin suppressed HCC recurrence and reduced macrophage infiltration in the postoperative liver. A.** Chemical structure of genipin.** B.** The schematic flowchart of the experimental procedure. **C&D.** Genipin dose-dependently (25 mg/kg and 50 mg/kg per 2 days) repressed the onset and progression of postoperative HCC recurrence as evidenced by luciferase signal intensity (n=5). **E.** The livers were dissected at the end of study. Genipin-treated mice showed less recurrence rate with smaller recurrent tumour sizes (n=5). **F.** H&E staining of the liver sections showed less recurrent foci in genipin-treated mice. Ki67 (proliferation marker) staining of the recurrent tumour sections showed that genipin treatment dose-dependently decreased the proliferating tumour cell number. **G&H.** Genipin exhibited minimal influence to mice body weight and histology of major organs revealed by H&E staining. **I.** Immune population profiling by FACs showing that genipin significantly decreased the CD11b^+^F4/80^+^ macrophage population in the postsurgical liver without influence on other immune populations. **J.** F4/80 staining on the recurrent tumors and peritumoral liver sections demonstrated that genipin significantly decreased F4/80^+^ macrophages in both recurrent tumour and peritumoral liver tissues. All experiments were performed in triplicate if without particular notice. *p < 0.05; **p < 0.01; ***p < 0.001.

A natural component genipin (Fig. 2A) was previously reported to suppress HCC in vivo in our previous studies. To systematically investigate the efficacy of genipin in preventing postoperative recurrence of HCC, we established an *in situ* HCC recurrence model in athymic nude mice according to previous publications with minor modifications 30. The solid tumour cubes derived from human HCC cells MHCC-97L were implanted into the left robe of the mouse livers. Two-week after implantation, a secondary operation was performed to completely incise the observable tumours from the liver. Luciferase signal was checked to confirm the complete incision of the hepatic tumours. Mice were given a different dose of genipin (0, 25, 50 mg/kg) via oral administration every other day, starting from 2 days before surgery (Fig. 2B). The HCC recurrence after tumour incision was checked by bioluminescence signal under live animal imager every 7 days (Fig. 2C), and significant delay of the tumour recurrence was observed in genipin-treated mice (Fig. 2D). By the end of study observation, mice receiving genipin treatment had less chance of developing early recurrence, as well as the smaller size of recurrent tumours (Fig. 2E). H&E staining identified the metastatic foci and inflammation in the postoperative liver, while genipin intervention significantly reduced the area and several recurrent foci in the liver, as well as reduced inflammatory cell infiltration (Fig. 2F). Further Ki67 staining showed that recurrent tumour cells from genipin-treated mice had reduced proliferating properties (Fig. 2F), confirming the suppressive effect of genipin on HCC recurrence. Genipin exhibited excellent biosafety profile, as evidenced by minimal body weight changes during treatment period and negligible damages to major organs (Fig. 2G&H).

To identify the possible immunomodulatory mechanism involved in the suppressive activity of genipin on the postoperative recurrence of HCC, we performed the immune cell phenotyping of the liver in genipin-treated athymic nude mice by flow cytometry. We observed that genipin intervention had minimal effect on the overall B cell population or the immunosuppressive myeloid population, but significantly suppressed the CD11b^+^F4/80^+^ macrophages population in the postoperative liver (Fig. 2I). Consistently, immunofluorescence staining confirmed the suppressive effect of genipin on macrophage infiltration to the postoperative adjacent liver and recurrent tumour tissues (Fig. 2J). Considering the immunodeficient background of the athymic nude mice, we further established an immunocompetent HCC orthotopic mice model by injecting 2×10^6^ Hepa 1-6 cells into the left lobe of C57BL/6 mice and incised the tumours as aforementioned. Genipin intervention on the C57BL/6 mice with Hepa 1-6 tumours showed minimal effect on the B cells, myeloid-derived suppressive cells and various T cell populations from the postoperative livers, but significantly suppressed the hepatic macrophages (Fig.S1). These findings suggest that genipin suppressed postoperative recurrence of HCC with inhibition of hepatic macrophage population.

### Reduced hepatic macrophage infiltration was responsible for the suppressive effect of genipin on HCC postoperative recurrence

**Figure 3:**
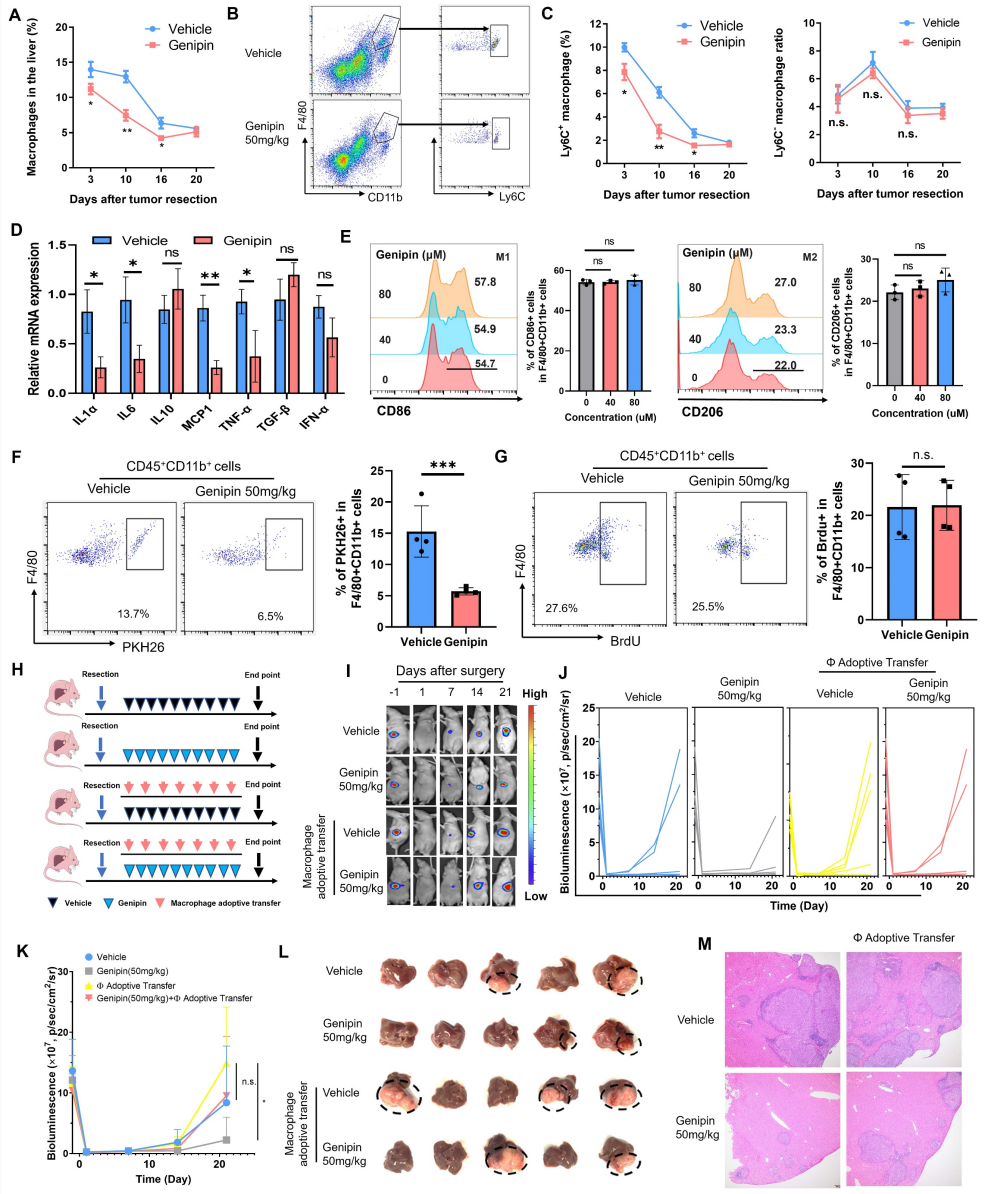
** Inhibition of hepatic infiltration of macrophages in the postoperative liver was responsible for the suppressive effect of genipin on HCC recurrence. A.** The time manner changes of total macrophage population (CD11b^+^F4/80^+^) in the postsurgical liver. **B.** representative gating on population of infiltrating and resident macrophages in the liver. **C.** The time manner changes of liver resident (Ly6C^-^CD11b^+^F4/80^+^) and inflammatory monocyte lineage (Ly6C^+^CD11b^+^F4/80^+^) infiltration in the postsurgical liver. Genipin significantly decreased monocyte-lineage macrophage infiltration while had minimal effects on resident macrophages. The infiltration of inflammatory monocyte lineage in 10 days postsurgical liver of mice treated with vehicle and genipin. **D.** The expression of pro-inflammatory cytokines in 10-days postoperative Ly6C^+^ macrophages was decreased by genipin. **E.** No influence in macrophage polarity was observed by the treatment of genipin.** F.** PKH26 labelling assay showed that genipin interfered with the infiltration process of PKH26+ macrophages. **G.** BrdU assay showed that genipin had negligible influence on the macrophage proliferation. **H.** Schematic diagram showing the treatment procedure.** I-K.** Luciferase signal intensity showed that macrophage adoptive transfer fuelled the progression of HCC recurrence and abolished the therapeutic effects of genipin (n=5). **L.** End-point recurrent tumours were dissected. **M.** H&E staining of the liver sections showed that less recurrent foci was observed in genipin treated mice while macrophage adoptive transfer abrogated this effect. All experiments were performed in triplicate if without particular notice. *p < 0.05; **p < 0.01; ***p < 0.001.

To decipher the action of genipin on the macrophages in the postoperative livers, we first delineated the dynamic changes of the macrophage population during the postoperative recurrence of HCC. The hepatic population of macrophages maintained at its high-level right after surgical operation, and gradually reduced along with the disease progression. Genipin treatment significantly suppressed the macrophage population in the liver at the early stage of post-surgery (Fig. 3A) Furthermore, genipin did not significantly change the resident CD11b^+^F4/80^+^Ly6C^-^ macrophage subpopulation in the postoperative liver but potently suppressed the infiltrating CD11b^+^F4/80^+^Ly6C^+^ macrophage subpopulation, which represent inflammatory monocyte-lineage macrophages (Fig. 3B&C). The inflammatory status of the 3 days postoperative Ly6C^+^ macrophages were measured by the expression of several pro-inflammatory markers, among which we observed that IL6, MCP-1 and TNF-α were upregulated upon surgical stress. While treatment of genipin mitigated the inflammation in Ly6C^+^ macrophages (Fig.S2). Similar results were observed in the 10 days postoperative Ly6C^+^ macrophages. Genipin repressed the expression of proinflammatory cytokines and chemokines without influencing anti-inflammatory cytokine expression (Fig. 3D). The anti-inflammation activity of genipin had marginal influence on the polarity of bone marrow-derived macrophages (BMDMs) as shown in Fig. 3E. To confirm the action of genipin on macrophage infiltration in the postoperative liver, we then injected PHK26-labelled BMDMs into the genipin-treated mice on the 10^th^ day of post-surgery. Mice with genipin treatment showed a significantly reduced population of PHK26^+^ BMDMs in the liver, confirming the ability of genipin in inhibiting macrophage infiltration into the postoperative liver (Fig. 3F). In addition, we found that genipin treatment had minimal effect on the DNA incorporation of BrdU injected into the postoperative mice (Fig. 3G), suggesting that genipin did not alter the proliferation of localised hepatic macrophages at the postoperative stage.

We then applied macrophage adoptive transfer to examine the essential role of reduced macrophage infiltration in the suppressive effect of genipin on HCC recurrence. BMDMs were injected every four days right after the surgical resection, along with genipin intervention (Fig. 3H). It was observed that adoptive transfer of BMDMs potently accelerated the onset of recurrent tumours growth in the liver of genipin-treated mice (Fig. 3I), and increased the rate of early HCC recurrence at the experimental endpoint (Fig. 3J&K). The size of recurrent tumours was increased upon macrophage adoptive transfer (Fig. 3L). Histologically, macrophage adoptive transfer was able to increase the intrahepatic metastatic foci in the postoperative liver of the genipin-treated mice (Fig. 3M). These findings confirmed the suppression of macrophage infiltration in the postoperative liver is responsible for the inhibitory effect of genipin on HCC recurrence.

### Genipin regulated PPAR signalling in the macrophages of postoperative liver by direct binding with PPARγ

**Figure 4:**
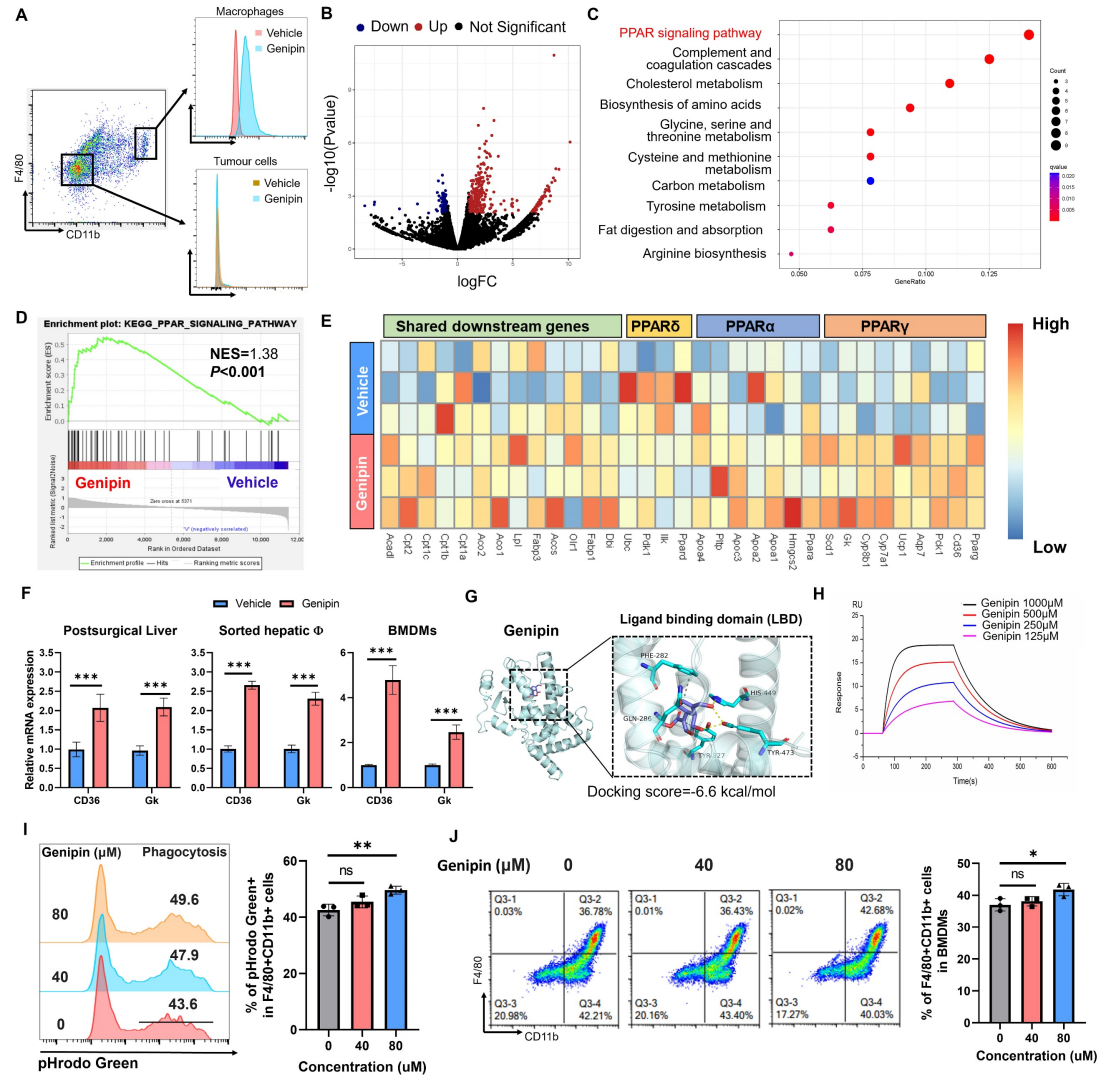
** Genipin binds PPARγ to active the downstream signalling in infiltrating macrophages in the postoperative liver. A.** Genipin could exclusively distribute to the hepatic macrophages (CD11b^+^F4/80^+^) as evidenced by the detection of autofluorescence of genipin. **B.** Volcano plot showing the differentially expressed genes between vehicle and genipin treated liver infiltrated macrophages. **C.** Dot plot showing the KEGG pathway enrichment results of differentially expressed genes between vehicle and genipin treated liver infiltrated macrophages. **D.** Gene set enrichment analysis (GSEA) analysis showing that PPAR signalling was activated in genipin treated liver macrophages. **E.** Heatmap showing the PPAR signalling associated gene expression profile. **F.** RT-qPCR results showed that genipin significantly triggered the upregulation of PPARγ downstream target genes expression in liver tissue, liver sorted macrophages and BMDMs. **G.** 3D structure of binding profile between PPARγ ligand binding domain and genipin is visualized and analysed by in silico molecular docking approach. Genipin exhibited comparable docking score in comparison with clinical approved PPARγ antagonist and agonist. **H.** surface plasmon resonance (SPR) analysis showing the binding affinity of genipin of different concentrations (125-1000 μM) over a PPARγ immobilized sensor chip. **I.** Genipin at the dose of 80 μM subtly promoted the phagocytosis of BMDMs. **J.** Genipin at the dose of 80 μM subtly promoted the differentiation of BMDMs. All experiments were performed in triplicate if without particular notice. ***p < 0.001.

To delineate the mechanisms of genipin in suppressing macrophage infiltration and HCC recurrence in the postoperative liver, we first identified the distribution profile of genipin in vivo. As genipin exposes autofluorescence signal, we dissected the liver and measured the single cell distribution of genipin by flow cytometry. Interestingly, we found genipin exclusively disseminates into the CD11b^+^F4/80^+^ cells, but not CD11b^-^F4/80^-^ cells, suggesting genipin has the direct effect on macrophages within postoperative liver (Fig. 4A). We therefore applied FACS to sort the CD11b^+^F4/80^+^ macrophages from the liver of genipin-treated mice three days after surgical resection, and the cells were subjected to RNA sequencing. It was found that genipin treatment regulated the expression of a series of genes in the sorted macrophages from the postoperative liver of mice (Fig. 4B). The lists of genes being upregulated or downregulated were then subjected to the DAVID web tool for the analysis of possible signalling pathway involved. PPAR signalling pathway was found to be the major related intracellular pathway regulated by genipin intervention on hepatic macrophages (Fig. 4C). Gene Set Enrichment Analysis (GSEA) revealed that the PPAR signalling in the macrophage from the postoperative liver of mice was activated upon genipin intervention (Fig. 4D).

The PPAR signalling includes three core transcriptional factors PPARα, PPARβ/δ and PPARγ. To investigate whether genipin selectively activates a particular PPAR, we extracted the PPAR downstream genes in the KEGG database and observed that genipin selectively upregulated PPARγ target genes like CD36 and Gk (Fig. 4E). The activation of PPAR signalling by genipin was then confirmed from the observation of up-regulation of transcriptional products of PPARγ upon genipin intervention in the postoperative liver, sorted macrophages as well as the in vitro culture of hepatocytes and BMDMs (Fig. 4F). We thus hypothesized that genipin directly interacts with PPARγ. Using Autodock prediction, we computationally confirmed that genipin could bind to PPARγ with a docking score at -6.6 kcal/mol, which was compatible with that of rosiglitazone and GW9662, the FDA-approved PPARγ agonist and antagonist (Fig. 4G, Fig.S3). Surface plasmon resonance assay confirmed the ex vivo binding between genipin and human PPARγ recombinant protein (Fig. 4H). PPARγ signalling plays an important role in the regulation of various biological process of macrophages. To investigate whether PPARγ activation by genipin will affect the phenotype of macrophages, we extracted BMDMs which was treated by series concentration of genipin. Our observation suggested that PPARγ activation by genipin subtly promoted the maturation and phagocytosis of macrophages (Fig. 4I&J), which echoed with previous study 31, 32. These observations together suggested that genipin regulated PPAR signalling in the macrophages of postoperative liver by direct binding with PPARγ.

### Activation of PPARγ by genipin conferred to inhibition of macrophage infiltration in the postoperative liver via suppressing CCR2 expression

**Figure 5:**
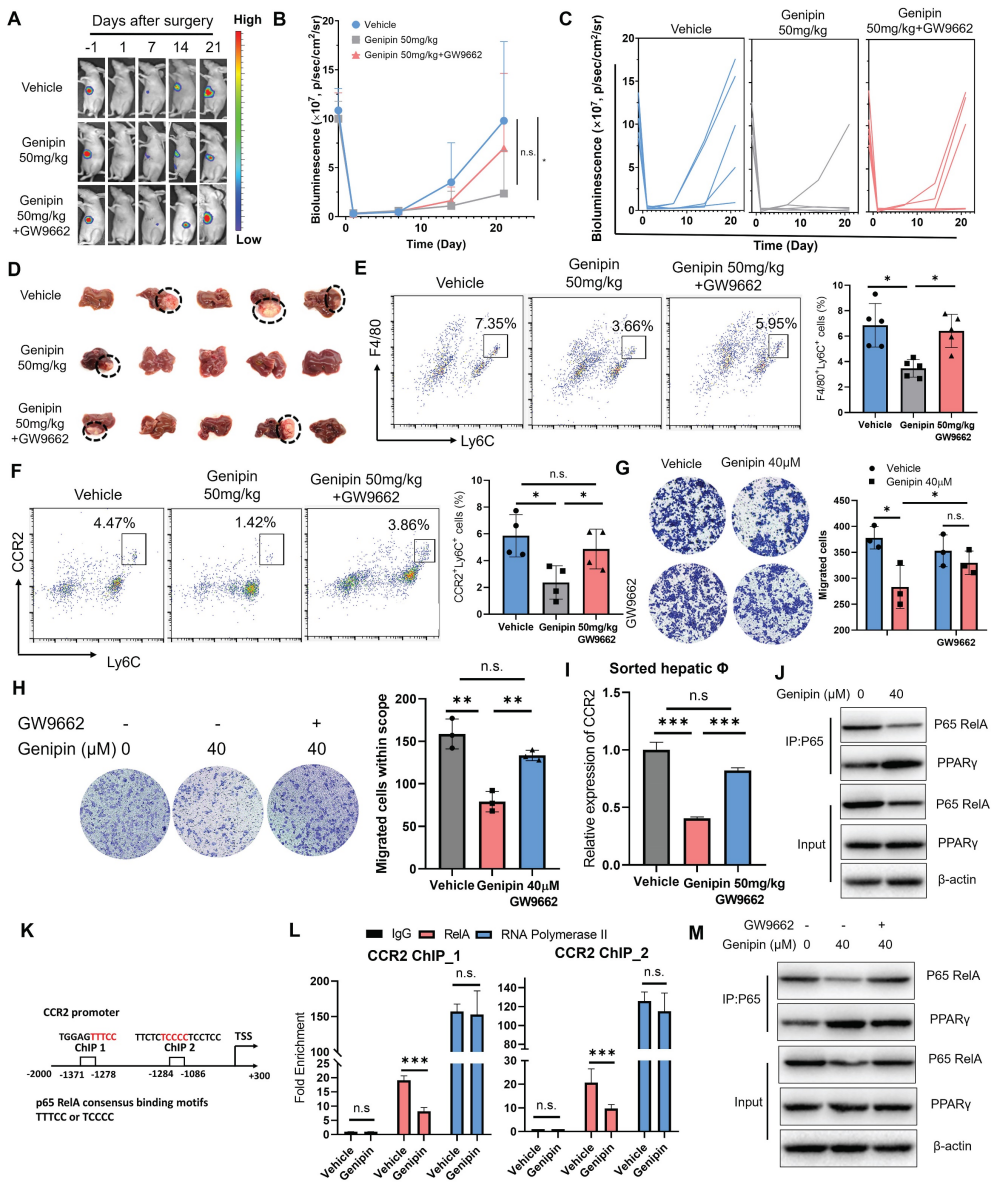
** Inhibition of PPARγ reversed the inhibitory effect of genipin on postoperative macrophage infiltration and HCC recurrence. A-D.** In vivo treatment of GW9662 abolished the therapeutic effects of genipin on delaying the onset and progression of postoperative HCC recurrence. **E&F.** In vivo treatment of GW9662 restored the population of CD11b^+^F4/80^+^Ly6C^+^ and Ly6C^+^CCR2^+^ macrophages in the postsurgical liver. **G.** GW9662 abolished the effects of genipin on macrophage migration towards CCL2.** H.** GW9662 treatment on AML-12 cells attenuated the chemo-attractive effects of AML-12 conditioned medium. **I.** Genipin treatment suppressed mRNA expression of CCR2 in sorted macrophages from postoperative liver, while PPARγ antagonism reversed this effect.** J.** Genipin treatment downregulated the protein level of p65 while increased the PPARγ-P65 interaction in BMDMs. **K.** The consensus binding motifs of p65 were found in the promoter region of CCR2 (-1086 to -1284, -1278 to -1371). **L.** Genipin treatment on BMDMs decreased the binding of p65 to CCR2 promoter region.** M.** GW9662 treatment attenuated genipin-induced p65 degradation and decreased PPARγ-p65 interaction in BMDMs. All experiments were performed in triplicate if without particular notice. *p < 0.05; **p < 0.01; ***p < 0.001.

To decipher the role of PPARγ activation in mediating the inhibitory effect of genipin on postoperative recurrence in HCC, we antagonised genipin-induced PPARγ activation by the presence of GW9662 33. The *in vivo* effect of PPARγ antagonist in reversing the inhibitory effect of genipin on the postoperative recurrence of HCC was examined. GW9662 was orally administrated to the HCC-bearing mice at the dose of 5 mg/kg. The presence of GW9662 in genipin-treated mice accelerated the onset of recurrent hepatic tumour growth (Fig. 5A) and increased the chance of early tumour recurrence in mice following surgical resection (Fig. 5B&C). The size of recurrent tumours at the experimental endpoints was larger in genipin-treated mice in the presence of GW9662 than that without GW9662 intervention (Fig. 5D). PPARγ inhibition by GW9662 in genipin-treated mice potently restored the abundance of CD11b^+^F4/80^+^Ly6C^+^ macrophages in the postoperative liver (Fig. 5E). A previous study documented that blocking CCR2 by antagonist efficiently thwarted HCC progression by inhibiting macrophage infiltration, underlining the important role of CCR2 in macrophage influx 21. Consistently with this report, we observed a significantly inhibition on CCR2 expression by genipin within CD11b^+^F4/80^+^Ly6C^+^ infiltrated macrophages, while PPARγ antagonism restored CCR2^+^ infiltrated macrophage population (Fig. 5F). CCR2 mediates chemoattractants-induced macrophage motility. We observed that supplementation of GW9662 to genipin-treated BMDMs restored its motility towards recombinant CCL2 in transwell assay (Fig. 5G). Similarly, treatment of genipin could significantly block BMDMs migration attracted by culture supernatant of murine hepatocyte cell line AML-12, which could be completely blocked by the presence of GW9662 (Fig. 5H). These findings suggested that activation of PPARγ by genipin is the core event involved in the suppression of postoperative recurrence of HCC.

The mRNA expression of CCR2 in the macrophages within postoperative liver of HCC mice was examined, which revealed that genipin treatment suppressed CCR2 expression at transcription level in the macrophages, while PPARγ antagonism restored CCR2 transcription (Fig. 5I). To investigate whether PPARγ directly enables genipin-mediated CCR2 downregulation, we conducted ChIP-qPCR assay and observed that PPARγ could not bind to the promoter region of CCR2 (Fig.S4), suggesting that genipin modulate CCR2 expression irrespective through PPARγ/CCR2 binding. Further screening for other potential transcriptional factors for CCR2 using the TRRUST database postulated that RelA was a common transcription factor of CCR2 and predominantly bind to the promoter region to initiate the transcriptional activation 34, 35. Previous studies suggested that the activated PPARγ inhibits the transcription activity of p65 RelA through direct binding 36. We, therefore, performed a co-immunoprecipitation assay with an antibody specific to NF-κB/p65 in genipin-treated hepatocytes and BMDMs and observed that genipin specifically increased the binding of PPARγ with p65 RelA, one of the subunits of NF-κB in both cells (Fig. 5J). Consequently, genipin treatment resulted in reduced p65 RelA protein expression, which may stem from PPARγ-mediated p65 degradation. Accordingly, genipin suppressed the association of p65 RelA on the promoter region of CCL2 and CCR2 respectively (Fig. 5K&L). The presence of PPARγ antagonist GW9662 on genipin-treated cells potently reduced the binding between PPARγ and p65 RelA and restored the p65 RelA expression (Fig. 5M). These data confirmed genipin as a PPARγ agonist that inhibits CCL2/CCR2 axis through transcriptional repression of p65 RelA activity.

### CCL2/CCR2 axis inhibition was involved as the microenvironmental event in the suppressive effect of genipin on postoperative macrophage infiltration

**Figure 6:**
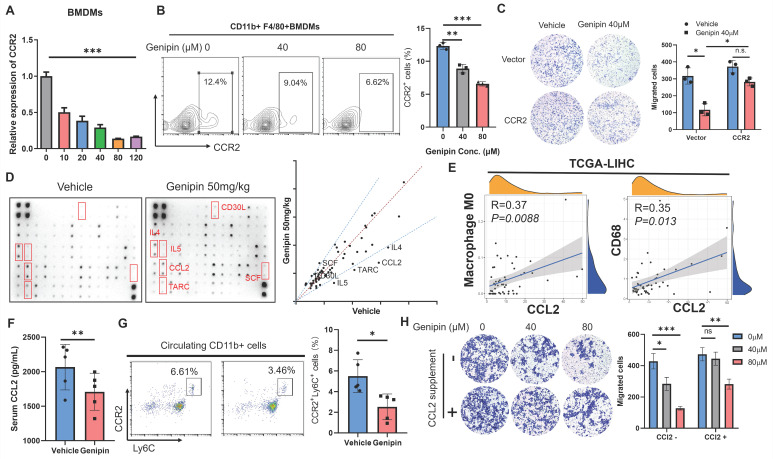
** Suppression of liver-derived CCL2 conferred to inhibitory effect of genipin on pro-inflammatory macrophage infiltration in the postoperative liver. A.** The expression of CCR2 in BMDMs was dose-dependently suppressed under genipin treatment (n=3). **B.** Genipin intervention on the BMDMs resulted in less CCR2^+^ populations. **C.** overexpression of CCR2 rescued BMDMs migration towards chemoattractant. **D.** Cytokine array revealed that CCL2 was among the four over 1.5-fold downregulated cytokines. **E.** Data mining in TCGA-LIHC dataset revealed that CCL2 in adjacent tissues significantly correlated with CD68 and M0 macrophage quantified by CIBERSORT algorithm (n=51). **F.** Genipin intervention suppressed circulating CCL2 level in mice bearing HCC. **G.** Genipin treatment significantly decreased Ly6C^+^CCR2^+^ monocyte population in the peripheral blood of postsurgical mice. **H.** supplementation of 50ng/ml CCL2 undermined the effects of genipin on macrophage motility towards chemoattractant. All experiments were performed in triplicate if without particular notice. *p < 0.05; **p < 0.01; ***p < 0.001.

To further confirm the functional role of CCR2 inhibition by genipin in mediating macrophage infiltration into the liver, we confirmed the expression changes of CCRs upon genipin treatment. As we observed genipin can directly interact with hepatic macrophage in vivo, we applied genipin to BMDMs directly in vitro. Treatment of genipin had minimal effects on the proliferation and survival of BMDMs (Fig.S5), but dose-dependently suppressed the mRNA expression as well as cell surface presentation of CCR2 (Fig. 6A&B). Furthermore, overexpression of CCR2 in macrophages significantly attenuated the inhibitory effect of genipin on macrophage motility, which had proven a direct link between CCR2 expression inhibition and genipin-induced suppression of macrophage infiltration in the postoperative liver (Fig. 6C).

Ly6C^+^CCR2^+^ monocyte population tends to be recruited by CCL2 and CCR2 along with CCL2 will be internalized through endocytosis pathway upon ligand-receptor interaction 37. To further explore the functional consequence of inhibition on CCR2-mediated macrophage infiltration by genipin, we performed a cytokine array to detect the pro-inflammatory cytokine changes in the postoperative liver of mice with or without genipin intervention (Fig. 6D). Using fold-change of 1.5 as a cut-off, we identified that CCL2 expression was potently downregulated in the postoperative liver upon genipin intervention (Fig. 6D). CCL2 is a cytokine produced during tissue injury and governs the chemotaxis of inflammatory monocytes. In the adjacent liver tissues of HCC patients (TCGA-LIHC), expression of CCL2 was positively correlated with naïve M0 macrophage (calculated by CIBERSORT algorithm) presentation and CD68 expression (Fig. 6E), indicating that genipin intervention suppressed CCL2 secretion from the hepatocytes in the postoperative liver, and thereby indirectly inhibited macrophage infiltration into the postoperative liver. Genipin treatment suppressed circulating level of CCL2 (Fig. 6F), and reduced the Ly6C^+^CCR2^+^ inflammatory monocyte population in the peripheral blood of mice after partial hepatectomy (Fig. 6G). Additionally, we supplemented CCL2 to the culture supernatant of hepatocytes and found that CCL2 replenishment significantly restored the BMDMs migration through the transwell membrane (Fig. 6H). These observations suggest that genipin exerted both direct and indirect inhibition on the macrophage infiltration to the postoperative liver via CCL2/CCR2 axis.

### The clinicopathological significance of PPARγ and macrophage chemotaxis in HCC recurrence

**Figure 7:**
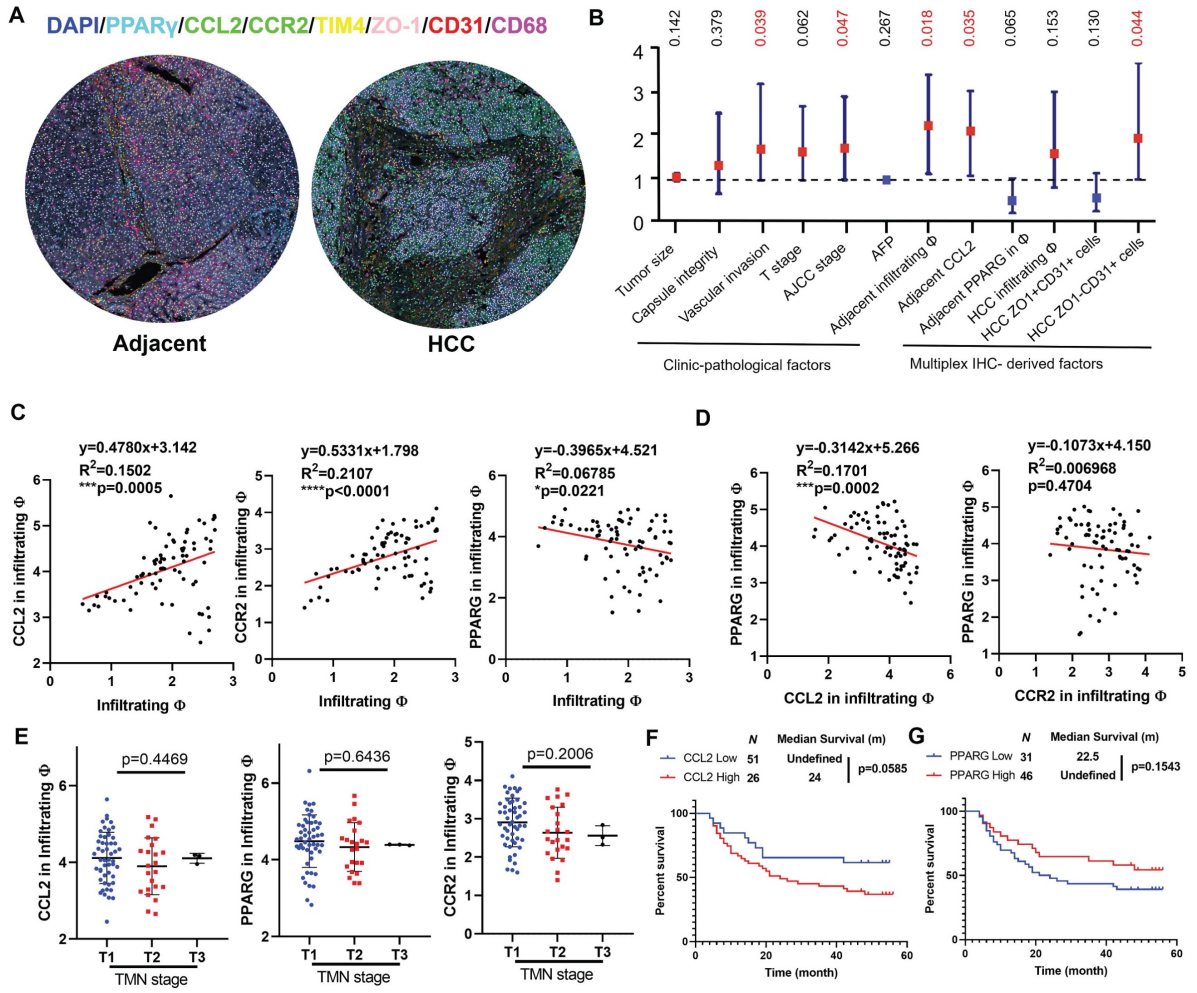
** Clinical prognosis value of CCL2/CCR2 axis in HCC patients with surgical operation. A.** Representative images of the multiplex IHC data analysis in tissue microarray. **B.** Forest plot showing the univariate Cox analysis results of clinic-pathological features and multiplex IHC derived factors in tissue microarray patient cohort. **C.** Pearson regression analysis showed that CCL2 and CCR expression in infiltrating macrophages was positively correlated with infiltrating macrophages in the adjacent liver, while PPARγ had the negative correlation. **D.** PPARγ expression had negative correlation with CCL2 expression but not CCR2 in the infiltrating macrophages of adjacent liver. **E.** CCL2, CCR2 or PPARγ expression in infiltrating macrophages of adjacent tissue was associated with TMN staging. **F.** Kaplan-Meier analysis showed that adjacent CCL2 expression marginally predicted poor prognosis on the recurrence-free survival of HCC patients after surgical resection. **G.** adjacent PPARγ expression had marginal correlation with better recurrence-free survival in HCC patients. *p < 0.05; **p < 0.01; ***p < 0.001.

To explore the clinical significance of the molecular targets identified in this study, we performed multiplex immunohistochemical staining on a tissue array from 78 patients containing HCC sections and their paired adjacent tissue sections. The whole slide image, antibody staining panel and multiplex IHC analysis workflow are presented in Fig.S6, while representative images of adjacent and HCC tissues were shown in Fig. 7A. We assigned the TIM4^+^CD68^+^ cells as resident macrophages, TIM4^-^CD68^+^ cells as infiltrating macrophages, ZO-1^+^CD31^+^ cells as vascular endothelial cells lining tight junction and ZO-1^-^CD31^+^ as leaky vascular endothelial cells. In HCC tissues, the infiltration of CD68^+^ macrophages showed negative correlation with recurrence-free survival (RFS), though only marginal statistical significance was obtained. The abundance of ZO-1^+^CD31^+^ cells was associated with better RFS while ZO-1^-^CD31^+^ cells were associated with worse RFS, suggesting that leaky vasculature predicts a higher chance of postoperative HCC recurrence. In adjacent tissues, infiltrating macrophages and CCL2 predicted worse RFS with statistical significance. The intensity of PPARγ within infiltrating macrophages was associated with better prognosis though without statistical significance, indicating that PPARγ activation in hepatic infiltrating macrophages potentially benefits HCC patients. Similar results were obtained in univariate cox analysis (Fig. 7B), in which vascular invasion, AJCC stage, infiltrating macrophages and CCL2 in adjacent tissues, and ZO-1^-^CD31^+^ cells were identified as negative prognostic factors in the patient cohort. Correlation analysis showed that CCL2 expression and CCR2 in the infiltrating macrophages were positively associated with infiltrating macrophages in the adjacent tissues, while PPARγ expression in the infiltrating macrophages had the negative correlation with macrophage infiltration in the adjacent tissue (Fig. 7C). Furthermore, expression of PPARγ had negative correlation with CCL2 in infiltrating macrophages, while the correlation between PPARγ and CCR2 expression in infiltrating macrophages was not significant, probably due to the CCR2 internalisation through endocytosis pathway upon ligand-receptor interaction of CCL2/CCR2 37 (Fig. 7D). CCL2, CCR2 or PPARγ expression in infiltrating macrophages of adjacent tissue was associated with TMN staging (Fig. 7E). Kaplan-Meier analysis showed that adjacent CCL2 expression marginally predicted poor prognosis on the recurrence-free survival of HCC patients after surgical resection (Fig. 7F), while adjacent PPARγ expression had marginal correlation with better recurrence-free survival in HCC patients (Fig. 7G). To conclude, this part of the study demonstrated that infiltrating macrophages in the postsurgical liver is a promising target for postoperative HCC recurrence and activating PPARγ potentially decrease chemotaxis-associated macrophages infiltration.

## Discussion

The high incidence of postoperative HCC recurrence is the main culprit of dismal prognosis after primary HCC resection. The early recurrence of HCC is mainly derived from preoperative intrahepatic metastasis or cancer cells disseminated into liver tissues during surgery. However, existing therapeutic methods including repetitive hepatectomy and adjuvant chemotherapy seem less effective on the early-developed micro-tumour foci. Hence, developing new targets to thwart the progression of early HCC recurrence is of great significance. Back a century ago, Marie and Clunet found that incomplete incision of primary tumour resulted in more frequent development of metastasis.^38^ Similarly, Tyzzer corroborated this phenomenon by reporting that wider metastasis was observed when the primary tumour was partially resected in comparison to those without tumour resection 39. A recent study suggested that perioperative inflammatory responses favoured the growth of distant tumours 40. These observations suggested that surgery may benefit the expansion of disseminated micro-metastasis by creating a postoperative liver microenvironment characterized by inflammation, regeneration-associated growth factor release and wound healing, in which the target for HCC recurrence may lie. In this study, we emphasized the role and clinical significance of monocyte-derived macrophages in the postoperative HCC recurrence and reported that genipin, a natural compound derived from *Gardenia jasminoides*, effectively suppressed the recurrence of HCC by inhibiting the postoperative influx of inflammatory macrophages via dual chemotaxis modulation.

The pro-tumour role of macrophages has been well established 41. But the exact role of the macrophage in the development of HCC recurrence remains a puzzle. Ren et al. reported that increased CD68 expression in adjacent liver tissues conferred longer disease-free survival in HCC patients, suggesting that macrophage may delay the onset of recurrence 42. In a different study, it was observed that higher expression of M-CSF, CD31 together with another macrophage marker CD163 in the peritumoral area signified worse recurrence-free survival 43. Besides, CCL2, the key chemoattractant of inflammatory macrophages, was reported to be upregulated in patients with recurrent HCC 44. Our TCGA data mining indicated that both CD68 and CCL2 in adjacent liver tissues were associated with better RFS in HCC patients. These contradictory reports may be attributed to the heterogeneity of hepatic macrophages, which includes liver resident macrophages and monocyte lineage macrophages which infiltrated from circulation. To distinguish these two liver-residing macrophage populations, we added a resident macrophage marker TIM4 in our multiplex IHC study on tissue array, and we found that an abundance of monocyte lineage macrophages rather than resident counterparts was significantly associated with a higher risk for recurrence. Besides, the expression of CCL2 was significantly correlated with monocyte-derived macrophages rather than resident macrophages and associated with better RFS, suggesting that CCL2 was a potential target for macrophage influx and HCC recurrence.

Previous study has shown that blocking CCL2/CCR2 by CCR2 antagonist could effectively inhibit inflammatory macrophage infiltration and reverse immunosuppressive HCC microenvironment 45. Transcriptomic analysis showed that genipin was a natural agonist to PPARγ and could activate PPARγ downstream genes in both macrophages and liver cells. Activation of PPAR signalling in macrophages is usually associated with enhanced lipid metabolism and anti-inflammation 46, 47. Yet it remains unknown if PPARs regulate macrophage infiltration. ChIP-qPCR results demonstrated that PPARγ does not directly regulate the CCL2/CCR2 expression. Intriguingly, it was reported that PPARγ could serve as an E3 ligase triggering the degradation of p65/RelA, which has been proved as the transcriptional factor for CCL2 48, 49. We validated this result by ChIP-qPCR. And it arose our interest in whether p65 also serves as a TF for CCR2, the transcriptional mechanism that is less reported 50. Animal TF database revealed that the consensus binding motifs of p65 were presented on the promoter region of CCR2 and ChIP-qPCR proved that p65 indeed binds to CCR2 promoter, which to the best of our knowledge has not been reported yet. Therefore, the modulation of CCL2/CCR2 chemotaxis of genipin was dependent on PPARγ mediated p65 degradation. Our subsequent study corroborated this conclusion by showing that the presence of PPARγ antagonist reversed in vitro and in vivo effects of genipin, suggesting the core role PPARγ in mediating the inhibiting effect of genipin on postoperative HCC recurrence.

In sum, in this study, we identified genipin as a natural and effective PPARγ agonist, which holds potential as a postoperative adjuvant candidate to delay the progression of HCC recurrence by modulating macrophage chemotaxis. Genipin is directly bound to PPARγ, inducing its association with cellular p65 that in turn led to p65 degradation and suppression of CCR2 transcription. This effect of genipin, therefore, conferred the suppression of CCL2/CCR2-mediated macrophage infiltration into the postoperative liver and tumour recurrence in HCC. Adoptive transfer of pro-inflammatory macrophages or the presence of PPARγ antagonist GW9662 completely abolished the inhibitory effect of genipin on postoperative macrophage chemotaxis as well as HCC recurrence. PPARγ expression was inversely associated with CCL2/CCR2 axis in hepatic macrophages and recurrence-free survival of HCC patients. Our study shed light on the potential application of genipin as an adjuvant treatment for HCC patients with a surgical operation.

## Supplementary Material

Supplementary figures and tables.

## References

[1] Siegel RL, Miller KD, Fuchs HE, Jemal A (2022). Cancer statistics, 2022. CA: a cancer journal for clinicians.

[2] Vogel A, Meyer T, Sapisochin G, Salem R, Saborowski A (2022). Hepatocellular carcinoma. Lancet.

[3] Niu ZS, Wang WH, Niu XJ (2022). Recent progress in molecular mechanisms of postoperative recurrence and metastasis of hepatocellular carcinoma. World journal of gastroenterology.

[4] Llovet J, Kelley R, Villanueva A, Singal A, Pikarsky E, Roayaie S Hepatocellular carcinoma Nat Rev Dis Primers. 2021; 7: 6. PubMed Article.

[5] Xu W, Guo R, Xu G, Sun L, Hu D, Xu H (2017). Management of intrahepatic recurrence after resection for hepatocellular carcinoma exceeding the barcelona clinic liver cancer criteria. Oncotarget.

[6] Imamura H, Matsuyama Y, Tanaka E, Ohkubo T, Hasegawa K, Miyagawa S (2003). Risk factors contributing to early and late phase intrahepatic recurrence of hepatocellular carcinoma after hepatectomy. J Hepatol.

[7] Xu XF, Xing H, Han J, Li ZL, Lau WY, Zhou YH (2019). Risk Factors, Patterns, and Outcomes of Late Recurrence After Liver Resection for Hepatocellular Carcinoma: A Multicenter Study From China. JAMA surgery.

[8] Portolani N, Coniglio A, Ghidoni S, Giovanelli M, Benetti A, Tiberio GA (2006). Early and late recurrence after liver resection for hepatocellular carcinoma: prognostic and therapeutic implications. Annals of surgery.

[9] Chan AC, Chan SC, Chok KS, Cheung TT, Chiu DW, Poon RT (2013). Treatment strategy for recurrent hepatocellular carcinoma: salvage transplantation, repeated resection, or radiofrequency ablation?. Liver Transplantation.

[10] Bruix J, Takayama T, Mazzaferro V, Chau GY, Yang J, Kudo M (2015). Adjuvant sorafenib for hepatocellular carcinoma after resection or ablation (STORM): a phase 3, randomised, double-blind, placebo-controlled trial. Lancet Oncol.

[11] Krall JA, Reinhardt F, Mercury OA, Pattabiraman DR, Brooks MW, Dougan M (2018). The systemic response to surgery triggers the outgrowth of distant immune-controlled tumors in mouse models of dormancy. Science translational medicine.

[12] Demicheli R, Retsky MW, Hrushesky WJ, Baum M, Gukas ID (2008). The effects of surgery on tumor growth: a century of investigations. Annals of oncology: official journal of the European Society for Medical Oncology.

[13] Sun Y, Wu L, Zhong Y, Zhou K, Hou Y, Wang Z (2021). Single-cell landscape of the ecosystem in early-relapse hepatocellular carcinoma. Cell.

[14] Gao Q, Wang XY, Qiu SJ, Yamato I, Sho M, Nakajima Y (2009). Overexpression of PD-L1 significantly associates with tumor aggressiveness and postoperative recurrence in human hepatocellular carcinoma. Clin Cancer Res.

[15] Yang Y, Sun M, Yao W, Wang F, Li X, Wang W (2020). Compound kushen injection relieves tumor-associated macrophage-mediated immunosuppression through TNFR1 and sensitizes hepatocellular carcinoma to sorafenib. J Immunother Cancer.

[16] Sharma A, Seow JJW, Dutertre CA, Pai R, Blériot C, Mishra A (2020). Onco-fetal Reprogramming of Endothelial Cells Drives Immunosuppressive Macrophages in Hepatocellular Carcinoma. Cell.

[17] Tacke F (2017). Targeting hepatic macrophages to treat liver diseases. J Hepatol.

[18] Liu XL, Li FQ, Liu LX, Li B, Zhou ZP (2013). TNF-α, HGF and macrophage in peritumoural liver tissue relate to major risk factors of HCC Recurrence. Hepato-gastroenterology.

[19] Ding T, Xu J, Wang F, Shi M, Zhang Y, Li S-P (2009). High tumor-infiltrating macrophage density predicts poor prognosis in patients with primary hepatocellular carcinoma after resection. Human pathology.

[20] Zhu X-D, Zhang J-B, Zhuang P-Y, Zhu H-G, Zhang W, Xiong Y-Q (2008). High expression of macrophage colony-stimulating factor in peritumoral liver tissue is associated with poor survival after curative resection of hepatocellular carcinoma. Journal of clinical oncology.

[21] Li X, Yao W, Yuan Y, Chen P, Li B, Li J (2017). Targeting of tumour-infiltrating macrophages via CCL2/CCR2 signalling as a therapeutic strategy against hepatocellular carcinoma. Gut.

[22] Feng Y-B, Luo W-Q, Zhu S-Q (2008). Explore new clinical application of Huanglian and corresponding compound prescriptions from their traditional use. Zhongguo Zhong yao za zhi= Zhongguo Zhongyao Zazhi= China Journal of Chinese Materia Medica.

[24] Wang Y, Zhao T, Deng Y, Hou L, Fan X, Lin L (2019). Genipin Ameliorates Carbon Tetrachloride-Induced Liver Injury in Mice via the Concomitant Inhibition of Inflammation and Induction of Autophagy. Oxid Med Cell Longev.

[25] Zhang A, Wang S, Zhang J, Wu H (2016). Genipin alleviates LPS-induced acute lung injury by inhibiting NF-κB and NLRP3 signaling pathways. Int Immunopharmacol.

[26] Wang N, Zhu M, Tsao SW, Man K, Zhang Z, Feng Y (2012). Up-regulation of TIMP-1 by genipin inhibits MMP-2 activities and suppresses the metastatic potential of human hepatocellular carcinoma. PloS one.

[27] Tan HY, Wang N, Tsao SW, Che CM, Yuen MF, Feng Y (2016). IRE1alpha inhibition by natural compound genipin on tumour associated macrophages reduces growth of hepatocellular carcinoma. Oncotarget.

[28] Wang N, Tan H-Y, Lu Y, Chan Y-T, Wang D, Guo W (2021). PIWIL1 governs the crosstalk of cancer cell metabolism and immunosuppressive microenvironment in hepatocellular carcinoma. Signal Transduction and Targeted Therapy.

[29] Surace M, DaCosta K, Huntley A, Zhao W, Bagnall C, Brown C (2019). Automated Multiplex Immunofluorescence Panel for Immuno-oncology Studies on Formalin-fixed Carcinoma Tissue Specimens. J Vis Exp.

[30] Feng YX, Wang T, Deng YZ, Yang P, Li JJ, Guan DX (2011). Sorafenib suppresses postsurgical recurrence and metastasis of hepatocellular carcinoma in an orthotopic mouse model. Hepatology.

[31] Okreglicka K, Iten I, Pohlmeier L, Onder L, Feng Q, Kurrer M (2021). PPARγ is essential for the development of bone marrow erythroblastic island macrophages and splenic red pulp macrophages. The Journal of experimental medicine.

[32] Majai G, Sarang Z, Csomós K, Zahuczky G, Fésüs L (2007). PPARgamma-dependent regulation of human macrophages in phagocytosis of apoptotic cells. Eur J Immunol.

[33] Omeragic A, Kara-Yacoubian N, Kelschenbach J, Sahin C, Cummins CL, Volsky DJ (2019). Peroxisome Proliferator-Activated Receptor-gamma agonists exhibit anti-inflammatory and antiviral effects in an EcoHIV mouse model. Sci Rep.

[34] Lawrence DM, Seth P, Durham L, Diaz F, Boursiquot R, Ransohoff RM (2006). Astrocyte differentiation selectively upregulates CCL2/monocyte chemoattractant protein-1 in cultured human brain-derived progenitor cells. Glia.

[35] Chen MF, Li YJ, Yang TL, Lou B, Xie XM (2009). Losartan inhibits monocytic adhesion induced by ADMA via downregulation of chemokine receptors in monocytes. Eur J Clin Pharmacol.

[36] Hou Y, Moreau F, Chadee K (2012). PPARγ is an E3 ligase that induces the degradation of NFκB/p65. Nat Commun.

[37] Lopez MG, Martínez AA, Lamaze C, Martínez-a C, Fischer T (2009). Inhibition of dynamin prevents CCL2-mediated endocytosis of CCR2 and activation of ERK1/2. Cellular signalling.

[38] Marie P, Clunet J (1910). Frequence des metastases viscerales chez les souris cancereuses apres ablation chirurgicale de leur tumeur. Bull Assoc Fr Etud Cancer.

[39] Tyzzer EE (1913). Factors in the production and growth of tumor metastases. The Journal of Medical Research.

[40] Krall JA, Reinhardt F, Mercury OA, Pattabiraman DR, Brooks MW, Dougan M (2018). The systemic response to surgery triggers the outgrowth of distant immune-controlled tumors in mouse models of dormancy. Sci Transl Med.

[41] Mantovani A, Marchesi F, Malesci A, Laghi L, Allavena P (2017). Tumour-associated macrophages as treatment targets in oncology. Nature reviews Clinical oncology.

[42] Ren CX, Leng RX, Fan YG, Pan HF, Li BZ, Wu CH (2017). Intratumoral and peritumoral expression of CD68 and CD206 in hepatocellular carcinoma and their prognostic value. Oncol Rep.

[43] Kono H, Fujii H, Furuya S, Hara M, Hirayama K, Akazawa Y (2016). Macrophage colony-stimulating factor expressed in non-cancer tissues provides predictive powers for recurrence in hepatocellular carcinoma. World J Gastroenterol.

[44] Zhou SL, Zhou ZJ, Hu ZQ, Huang XW, Wang Z, Chen EB (2016). Tumor-Associated Neutrophils Recruit Macrophages and T-Regulatory Cells to Promote Progression of Hepatocellular Carcinoma and Resistance to Sorafenib. Gastroenterology.

[45] Li X, Yao W, Yuan Y, Chen P, Li B, Li J (2017). Targeting of tumour-infiltrating macrophages via CCL2/CCR2 signalling as a therapeutic strategy against hepatocellular carcinoma. Gut.

[46] Wang X, Ji Y, Feng P, Liu R, Li G, Zheng J (2021). The m6A Reader IGF2BP2 Regulates Macrophage Phenotypic Activation and Inflammatory Diseases by Stabilizing TSC1 and PPARγ. Adv Sci (Weinh).

[47] Liu S, Zhang H, Li Y, Zhang Y, Bian Y, Zeng Y (2021). S100A4 enhances protumor macrophage polarization by control of PPAR-γ-dependent induction of fatty acid oxidation. J Immunother Cancer.

[48] Ueda A, Ishigatsubo Y, Okubo T, Yoshimura T (1997). Transcriptional regulation of the human monocyte chemoattractant protein-1 gene. Cooperation of two NF-kappaB sites and NF-kappaB/Rel subunit specificity. J Biol Chem.

[49] Mas S, Martínez-Pinna R, Martín-Ventura JL, Pérez R, Gomez-Garre D, Ortiz A (2010). Local non-esterified fatty acids correlate with inflammation in atheroma plaques of patients with type 2 diabetes. Diabetes.

[50] Jung H, Miller RJ (2008). Activation of the nuclear factor of activated T-cells (NFAT) mediates upregulation of CCR2 chemokine receptors in dorsal root ganglion (DRG) neurons: a possible mechanism for activity-dependent transcription in DRG neurons in association with neuropathic pain. Mol Cell Neurosci.

