# Supplementary material for: Genipin-activating PPARγ impedes CCR2-mediated macrophage infiltration into postoperative liver to suppress recurrence of hepatocellular carcinoma
Source: Int J Biol Sci. 2023 Oct 16;19(16):5257–74. doi: 10.7150/ijbs.87327 (PMC10620825; doi:10.7150/ijbs.87327)
Supplement: Supplementary file 1 — Supplementary figures and tables. [file ijbsv19p5257s1.pdf]

Supporting information for

Genipin-activating PPARγ impedes CCR2-mediated macrophage infiltration into postoperative liver to suppress recurrence of hepatocellular carcinoma

Junyu Wu[1], Yau-Tuen Chan[1], Yuanjun Lu[1], Zixin Feng[1], Hongchao Yuan[1], Xiaoyu Xu[1], Lin Xu[1], Cheng Zhang[1], Yibin Feng[1], Hor-Yue Tan[2,*], Ning Wang[1,3,*]

1. School of Chinese Medicine, The University of Hong Kong, Hong Kong S.A.R., China

2. Centre for Chinese Herbal Medicine Drug Development, School of Chinese Medicine, Hong Kong Baptist University, Hong Kong S.A.R., China

3. Department of Chinese Medicine, the University of Hong Kong-Shenzhen Hospital (HKU-SZH), Shenzhen, China

Supporting Figure S1
Supporting Figure S2
Supporting Figure S3
Supporting Figure S4
Supporting Figure S5
Supporting Figure S6

Supporting Table S1
Supporting Table S2
Supporting Table S3
Supporting Table S4

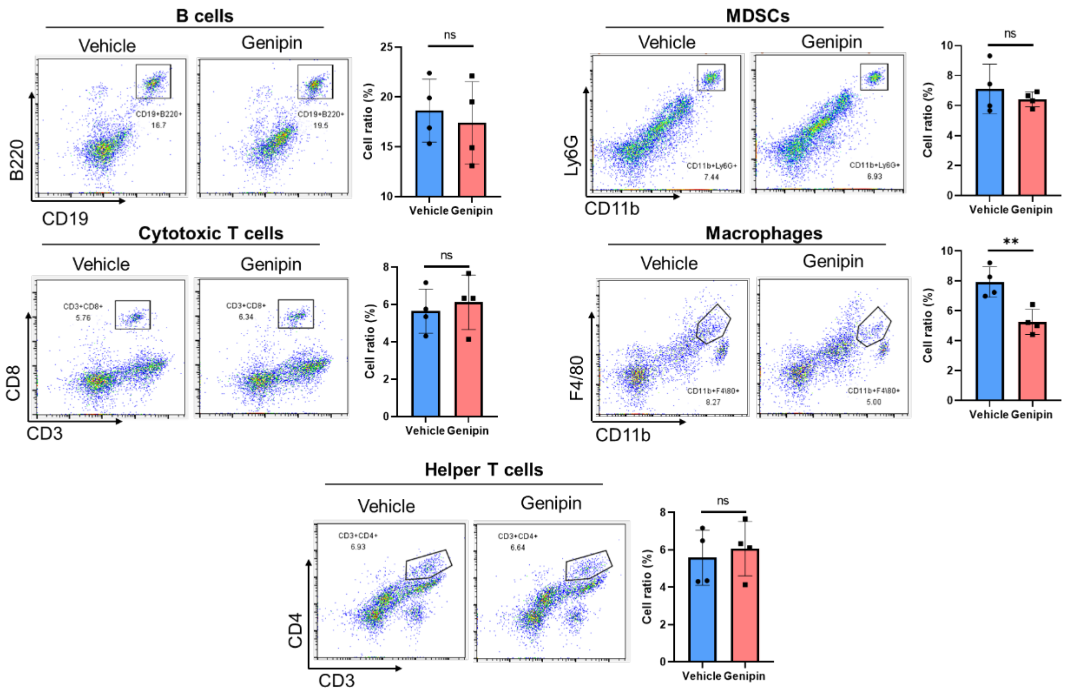

Figure S1. Flow cytometry results showing that treatment of genipin significantly decreased the infiltration of CD11b$^+$F4/80$^+$ to the postsurgical liver without influence on other innate immune populations in C57/BL/J mice bearing HCC tumours. ***P*<0.01.

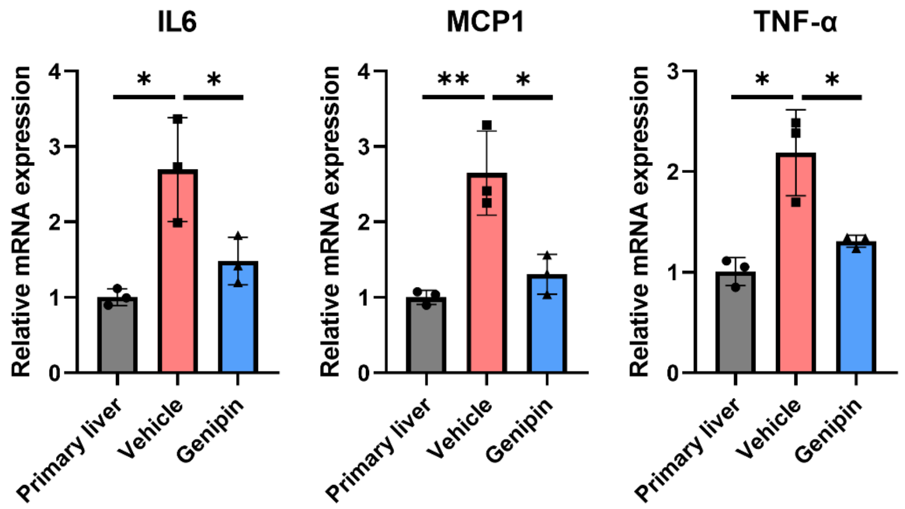

Figure S2. Relative expression of pro-inflammatory cytokines in preoperative and 3-days postoperative Ly6C+ macrophages sorted from vehicle and genipin treated mice livers. **P*<0.05, ***P*<0.01.

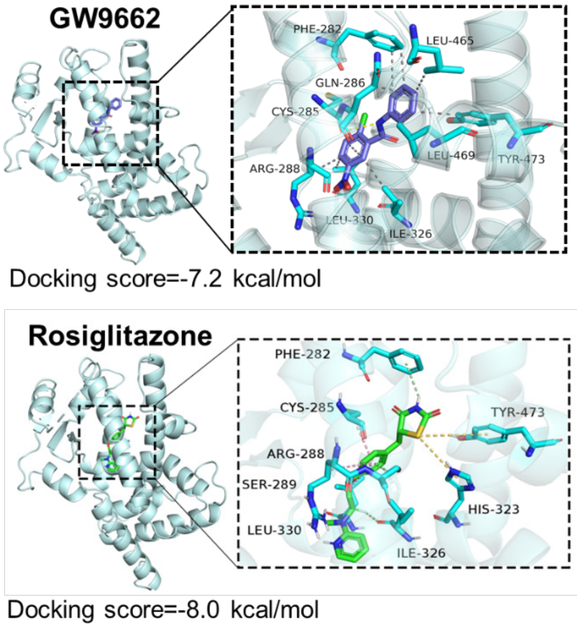

Figure S3 3D structure of binding profile between PPARγ ligand binding domain and GW9662 and Rosiglitazone is visualized and analysed by in silico molecular docking approach. GW9662 and Rosiglitazone exhibited comparable docking score.

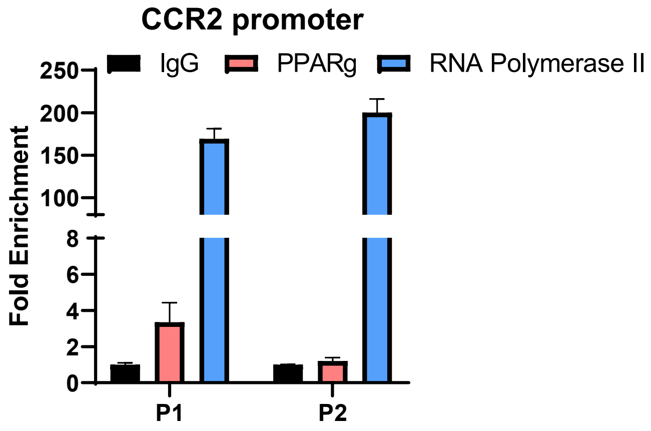

Figure S4 ChIP-qPCR analysis results showed that PPARγ couldn't bind to the promoter region of CCR2.

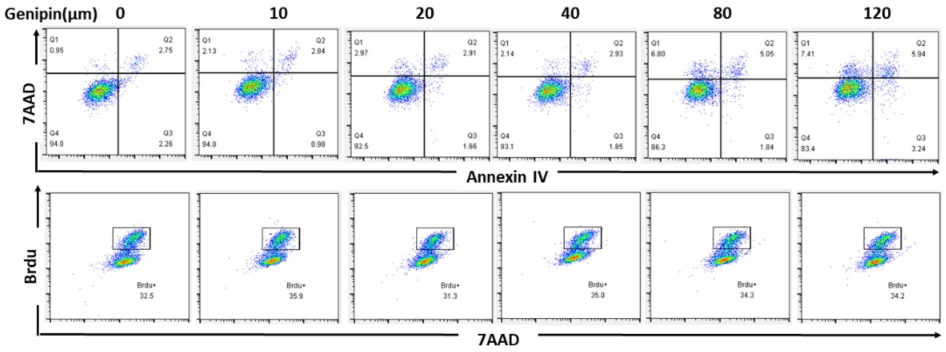

Figure S5 Cell apoptosis and BrdU assay showed that genipin treatment had little effects on the viability and proliferation of BMDMs.

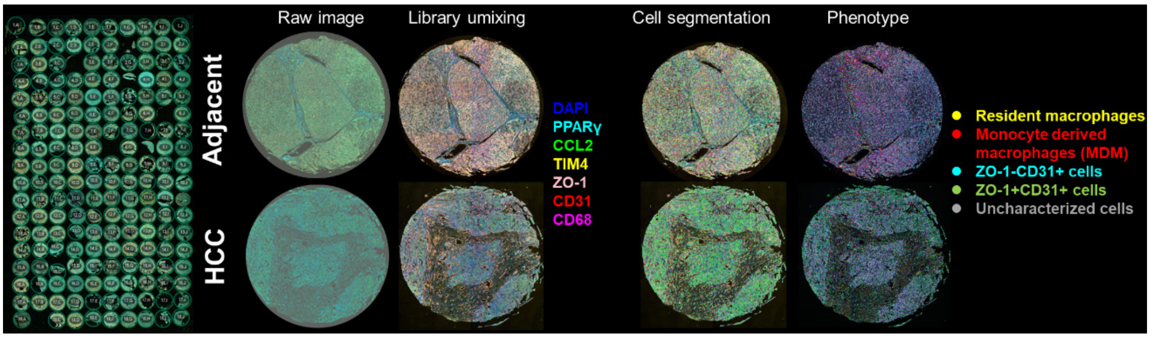

Figure S6 Overall workflow of the multiplex IHC data analysis in tissue microarray.

Supporting Table S1 List of antibodies used in flow cytometry and multiplex immunohistochemistry.

**Target protein**	**Clone**	**Brand**	**Cat No.**
CD3	17A2	BioLegend	100214
CD4	GK1.5	BioLegend	100406
CD8	SK1	BioLegend	344714
CD16/32	93	BioLegend	101302
CD11b	M1/70	BioLegend	101223
CD11c	N418	BioLegend	117325
CD86	Bu63	BioLegend	374203
CD206	C068C2	BioLegend	141739
F4/80	BM8	BioLegend	123108
Ly6G	1A8-Ly6g	Invitrogen	11-9668-82
Ly6C	HK1.4	BioLegend	128018
CCR2	SA203G11	BioLegend	150607
B220	RA3-6B2	BioLegend	103207
Brdu	RUO	BD Bioscience	559619
CCL2	Polyclonal	Abclonal	A7277
PPARγ	Polyclonal	Abclonal	A11183
TIM4	Polyclonal	Abcam	ab47637
CD31	Polyclonal	Abcam	ab28364
CD68	Kp1	Invitrogen	MA5-13473
ZO-1	Polyclonal	Abcam	ab59720

Supporting Table S2 Primers for qRT-PCR analysis

	**Forward primer**	**Reverse Primer**
**mCCR2**	ATCCACGGCATACTATCAACATC	CAAGGCTCACCATCATCGTAG
**mPPARg**	TCGCTGATGCACTGCCTATG	GAGAGGTCCACAGAGCTGATT
**mCD36**	ATGGGCTGTGATCGGAACTG	GTCTTCCCAATAAGCATGTCTCC
**mGk**	TGAACCTGAGGATTTGTCAGC	CCATGTGGAGTAACGGATTTCG

**mCCR2-ChIP1**	TTTGTGGGTCATGCCAAGGT	GTCCCCTTTCTGCCCATCTC
**mCCR2-ChIP2**	AGGGGACACACGAAGAACAC	GTCAGGTGTGCAGTGGATGA
**IL6**	TAGTCCTTCCTACCCCAATTTCC	TTGGTCCTTAGCCACTCCTTC
**IL10**	GCTCTTACTGACTGGCATGAG	CGCAGCTCTAGGAGCATGTG
**IL-1α**	CGAAGACTACAGTTCTGCCATT	GACGTTTCAGAGGTTCTCAGAG
**MCP1**	TTAAAAACCTGGATCGGAACCAA	GCATTAGCTTCAGATTTACGGGT
**TNF-α**	CCCTCACACTCAGATCATCTTCT	GCTACGACGTGGGCTACAG
**TGF-β**	CTCCCGTGGCTTCTAGTGC	GCCTTAGTTTGGACAGGATCTG

Supporting Table S3. Clinicopathological factors of tissue miacroarray cohort

**Clinicopathological factors**
Age (≥ 60 y, n = 20; < 60 y, n = 70)
Sex (male, n = 76; female, n = 16)
Tumor size (≥ 3.5 cm, n =64; < 3.5 cm, n = 26)
Recurrence (Yes, n=53; no, n=37)
HBsAg (positive, n = 77; negative, n = 13)
HBcAb (positive, n=80; negative, n=8; N.A, n=2)
Cirrhosis (Yes, n=78, no, n=12)
